# Therapeutic Efficacy of Antioxidants in Ameliorating Obesity Phenotype and Associated Comorbidities

**DOI:** 10.3389/fphar.2020.01234

**Published:** 2020-08-13

**Authors:** Steven Tun, Caleb James Spainhower, Cameron Lee Cottrill, Hari Vishal Lakhani, Sneha S. Pillai, Anum Dilip, Hibba Chaudhry, Joseph I. Shapiro, Komal Sodhi

**Affiliations:** Departments of Medicine, Surgery and Biomedical Sciences, Marshall University Joan C. Edwards School of Medicine, Huntington, WV, United States

**Keywords:** obesity, oxidative stress, antioxidants, adipocytes, cardiovascular disease, diabetes

## Abstract

Obesity has been a worldwide epidemic for decades. Despite the abundant increase in knowledge regarding the etiology and pathogenesis of obesity, the prevalence continues to rise with estimates predicting considerably higher numbers by the year 2030. Obesity is characterized by an abnormal lipid accumulation, however, the physiological consequences of obesity are far more concerning. The development of the obesity phenotype constitutes dramatic alterations in adipocytes, along with several other cellular mechanisms which causes substantial increase in systemic oxidative stress mediated by reactive oxygen species (ROS). These alterations promote a chronic state of inflammation in the body caused by the redox imbalance. Together, the systemic oxidative stress and chronic inflammation plays a vital role in maintaining the obese state and exacerbating onset of cardiovascular complications, Type II diabetes mellitus, dyslipidemia, non-alcoholic steatohepatitis, and other conditions where obesity has been linked as a significant risk factor. Because of the apparent role of oxidative stress in the pathogenesis of obesity, there has been a growing interest in attenuating the pro-oxidant state in obesity. Hence, this review aims to highlight the therapeutic role of antioxidants, agents that negate pro-oxidant state of cells, in ameliorating obesity and associated comorbidities. More specifically, this review will explore how various antioxidants target unique and diverse pathways to exhibit an antioxidant defense mechanism.

## Introduction

Chronic state of obesity is an ever-growing health concern burdening millions of individuals worldwide. Characterized physiologically as the accumulation of excess body fat, a substantial difficulty that comes with determining treatment and prevention of the disease is the multifactorial origin of the etiology (Hruby and Hu, 2015). The complexity of the etiology has led to obesity being one of the most uncontrollable disease epidemics of the last four decades. Since 1980, the prevalence of worldwide obesity has doubled with approximately 39% of people being overweight and 13% obese in 2014, according the World Health Organization. Further estimations indicate that by 2030, the worldwide obesity could reach 20% (Hruby and Hu, 2015; Mohammed et al., 2018). What is more worrisome is the increase of early onset obesity in children where obesity and potential comorbidities persist into adulthood in approximately 50% of cases versus 10% for children without obesity (Fruh, 2017). The stark incline of prevalence across all age groups is exceedingly concerning when considering the mortality associated with obesity. Across all age groups, according to compiled data of 19 cohort studies and 1.46 million participating individuals, mortality rate generally increased significantly in association with increased body mass index (BMI) (Berrington de Gonzalez et al., 2010). On average, obesity can lead to as much as a 5–10 year decrease on life expectancy (Kuk et al., 2011; Fruh, 2017).

Obesity has been known to be a cause of wide range of complications due to several underlying pathophysiological processes. Comorbidities of obesity are diverse and can occur in many bodily systems including cardiovascular complications, type 2 diabetes mellitus (T2DM), non-alcoholic steatohepatitis (NASH), metabolic syndrome, and several other lesser known morbidities (Sodhi et al., 2015; Srikanthan et al., 2016; Fruh, 2017; Lakhani et al., 2018; Lauby-Secretan et al., 2019). The trigger for obesity and associated comorbidities is intricately linked with an increase in reactive oxygen species (ROS) and subsequent oxidative stress. Several primary sources of endogenous intracellular ROS have been implicated including cellular mitochondria, endoplasmic reticulum (ER) stress, activation of oxidative stress pathways and upregulated activity of NADPH oxidase (NOX) (Bhatti et al., 2017). This causes a state of redox imbalance, where the pro-oxidants are excessively produced and the antioxidant defense mechanisms are diminished, facilitating a state of chronic inflammation (Fonseca-Alaniz et al., 2007; Wensveen et al., 2015). The interlinking relation between systemic redox imbalance and release of inflammatory mediators creates an inflammatory milieu affecting the regulation of metabolic pathways, consequences of which can lead to impaired physiological functions in obesity and associated comorbidities.

Due to the modulation of redox mechanisms in obesity, evidence suggests that a multimodal approach to treatment including diet changes, exercise, and medical treatments may be successful in curbing oxidant stress (Thompson et al., 2007; Achkasov et al., 2016). One such considerable approach against obesity is to counter the cellular pro-oxidant state by ameliorate the excessive production of ROS and subsequent oxidative stress. Antioxidants may present a viable therapeutic target to help ameliorate the negative effects of obesity and oxidative stress on the physiological systems of the body. The cumulative line of evidence suggests that antioxidants may modulate dynamic cellular targets and processes to improve the redox imbalance in obesity. The mechanisms involved in the regulation of cellular redox present increasing importance of unraveling the potential of endogenous and exogenous antioxidants in ameliorating obesity associated phenotype. Hence, this review aims to independently uncover the potential of several antioxidants in ameliorating obesity phenotype and associated comorbidities. Specifically, this review presents evidence from past literature in elucidating the role of antioxidants in improving antioxidant defense mechanism by manipulating localized redox signaling pathways in an obese state.

## Oxidative Stress in Obesity and Associated Comorbidities

The complex relationship between obesity and the associated comorbidities involves, first, the dysregulation of the vital communication system that adipocytes have within the body. Compared to the smaller, healthy adipocytes, the influence of obesity is drastic causing a transition to a subset of large, dysfunctional adipocytes (Lakhani et al., 2018). These larger adipocytes suffer from poor regulation mechanisms that disrupt the normal signaling functions that adipocytes play (Haczeyni et al., 2018). There are over 50 known adipokines that are released as signaling molecules from adipocytes including leptin, adiponectin, multiple interleukins, and TNF-α to name a few (Stolarczyk, 2017). In the obese model, the balance of these signaling adipokines are significantly disrupted. Pro-inflammatory adipokines (IL-6, TNF-α, MCP-1) and leptin, associated with the maintenance of the obese state, are elevated during the obese state whereas adiponectin, which plays an important role in insulin sensitivity, is decreased, connecting its role to insulin resistance and T2DM (Friedman and Halaas, 1998; Sirico et al., 2018). The increased levels of these pro-inflammatory adipokines place the body into a perpetual state of inflammation, or chronic inflammation. During the onset of obesity, this added inflammatory burden and subsequent adipocyte turnover/remodel to account for the increased fat accumulation is destructive to the cellular environment. Macrophages, which are recruited to aid in the adipocyte turnover, have also been implicated with obesity to potentiate the subsequent negative effects (Kuroda and Sakaue, 2017).

Following the stark transition to the state of chronic inflammation, the physiological environment quickly becomes burdened with oxidative stress in the form of increased ROS and oxidative radicals. One particular cellular component of concern following initial increase in ROS is at the mitochondrial level. Mitochondria, when functioning healthily, is already well-known as a major generator of ROS in the body (Oyewole and Birch-Machin, 2015). Invasion of macrophages to remodeling adipose tissue breaks down older adipocytes which leads to release of lipid components, inflammatory signals, and ROS (Lee et al., 2010). The continued release of inflammatory signals and ROS from this cycle of apoptosis and remodeling has a harmful effect on the mitochondria to exacerbate the inflammation and oxidative stress (Dela Cruz and Kang, 2018). ROS plays an imperative role in facilitating transition of healthy mitochondria to dysfunctional mitochondria. These dysfunctional mitochondria and increased ROS impair the Krebs cycle and respiratory chain (de Mello et al., 2018). The dysfunctional mitochondria produce abnormally high amounts of ROS that create a vicious positive feedback effect on the functional status of the mitochondria (Wang et al., 2013; de Mello et al., 2018). Although the chronic inflammation induces the state of oxidative stress, dysfunctional mitochondria are vital for maintaining the diseased state. Furthermore, brown adipose tissue (BAT) is a mitochondria rich tissue with high oxidative capacity which have been implicated in the process of adaptive thermogenesis (Lettieri-Barbato, 2019). The production of mitochondrial ROS under thermogenesis is important to maintain the bodily homeostasis, and this process is finely controlled through feedback mechanism. However, in a diseased metabolic condition, there is a shift in the redox state causing more oxidative stress during thermogenesis. This shift leads to altered expression of markers associated with thermogenesis causing an alteration in the adipocyte phenotype (Lettieri-Barbato, 2019). Studies have demonstrated a causal relationship between mitochondrial ROS and thermogenesis using the mitochondria-targeted antioxidant MitoQ, which efficiently ameliorates lipid peroxides and superoxides *in vivo* (Rodriguez-Cuenca et al., 2010). It is important to note that the redox changes occurring in BAT upon thermogenesis are dynamic, reversible and adapted to by antioxidant pathways (Chouchani et al., 2017).

This perpetual state of oxidative stress and inflammation is what links obesity to its associated comorbidities. The detrimental role that this diseased state plays on the cardiovascular system is substantial. Chronic inflammation has a negative effect on cardiovascular tissue contributing to the associated cardiovascular disease (CVD) and arteriosclerosis (Libby, 2006). With respect to obesity, the chronic, low-grade inflammation and oxidative from obesity places the cardiovascular system at an increased risk for developing plaques in blood vessels and cardiac remodeling (Kachur et al., 2017). Two studies, one done clinically with patients and the other in mice on high-fat diet, have shown that increased pressure in the heart stimulated an increase in function of NADPH oxidase 4 (NOX4), primarily located in the mitochondria of cardiac myocytes (Kuroda et al., 2010; Munzel et al., 2017). Under states of stress, NOX4 produces superoxide (O_2_^−^) to exacerbate the oxidative stress of the cardiovascular system (Kuroda et al., 2010). The inflammatory signals released by adipocytes also contribute to altering the physiology of the heart in obesity. Adiponectin, downregulated in obesity, plays a protective role in the heart by stimulating endothelial nitric oxide synthase (eNOS) to maintain healthy vascular tone. Conversely, the rising leptin levels in the heart positively correlate with coronary artery disease, left ventricular hypertrophy, stroke, and myocardial infarction (Soderberg et al., 1999; Wallace et al., 2001; Chait and den Hartigh, 2020). Along with pro-inflammatory circulating signals, a transcription factor, NF-*κ*B, is a product of chronic inflammation in cardiovascular tissue and is a useful clinical indicator for obesity-related inflammation (de Almeida et al., 2020).

**Figure 1:**
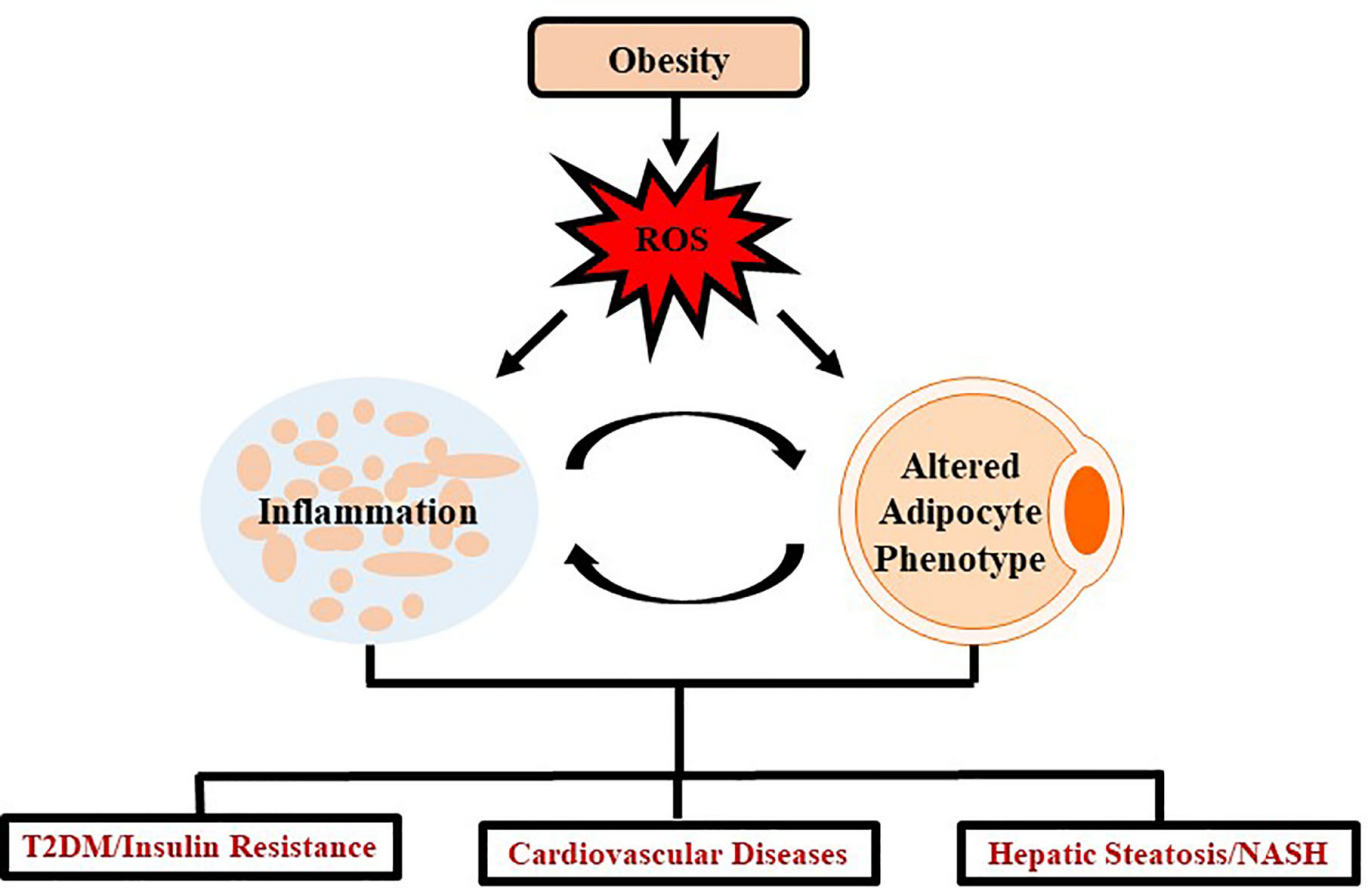
Schematic representation demonstrating impact of oxidative stress in obesity. Obesity mediates excessive production of ROS which leads to chronic inflammation and altered adipocyte phenotype which further induces several systemic changes causing oxidative stress. Such oxidative stress leads to the development and progression of chronic diseases such as T2DM, cardiovascular diseases and/or NASH.

Insulin resistance and T2DM are also example of diseases extensively linked as a comorbidity of obesity. A few of the prominent inflammatory adipokines associated with decreasing insulin sensitivity in tissues are IL-6 and TNF-α (Stolarczyk, 2017). In addition, IL-10 and adiponectin, two adipokines that promote insulin sensitivity are decreased in the obese state (Stolarczyk, 2017). Meanwhile, oxidative stress promotes a diabetic state by disrupting certain metabolic pathways such as inhibiting G-3-P dehydrogenase, stimulating buildup of G-3-P, and up-regulating glycolytic, hexosamine, advanced glycation end-product (AGE), protein kinase C, and polyl pathways (Ighodaro, 2018). Despite the specific mechanisms not being completely understood, the primary method by which obesity links to T2DM is through insulin resistance (Burhans et al., 2018). One proposed mechanism observed over multiple studies (but not all) is that as adipogenesis takes place, the adipocytes become dysregulated and release a larger amount of free fatty acids (FFA) into circulation (Mittendorfer et al., 2009). The FFA-mediated insulin resistance involves the products of oxidation of FFA in tissues which exacerbates already impaired glucose metabolism (Randle et al., 1965). Several *in vitro* studies have revealed a secondary mechanism linking the adipokine, TNF-α, to insulin resistance by regulating ceramide synthesis (Grigsby and Dobrowsky, 2001; Hernandez-Corbacho et al., 2015). Oxidative stress and inflammation resulting from obesity is a systemic problem impacting several regions of the body through similar mechanisms as seen in T2DM and cardiovascular complications (**Figure 1**).

## Antioxidants in Ameliorating Obesity and Associated Comorbidities

### Peroxiredoxin

Peroxiredoxins (Prxs) are a family of peroxidase enzymes that have a highly conserved function to reduce hazardous peroxides in the body. There are a few different subtypes within the Prx family, but the mechanism of action of the class as a whole is mediated through a highly conserved peroxidatic cysteine residue (C_P_) (Hall et al., 2009). Despite the fact that the catalytic function of all Prxs is conserved involving oxidation of the C_P_ by the peroxide substrate to sulfenic acid, the recycling of the sulfenic acid back to thiol is the defining feature of the three classes of Prxs: typical 2-Cys, atypical 2-Cys, and 1-Cys Prxs (Wood et al., 2003; Rhee, 2016). In the mammalian system, there are six known peroxiredoxin subtypes including PrxI-IV (typical 2-Cys), PrxV (atypical 2-Cys), and PrxVI (1-Cys) (Perkins et al., 2015).

Prxs are widespread across the body, and the subtypes are localized to several different cellular compartments to play a universal role against oxidative stress. PrxI and PrxII are both found predominantly in the cytosol or nuclei of cells with PrxII playing a vital role in protecting erythrocytes (Rhee et al., 2012). PrxIII (typical 2-Cys) and PrxV (atypical 2-Cys) are both found in the mitochondria where they are the primary antioxidant defense mechanism against the most abundant producer of ROS. PrxV, however, has also been found in peroxisomes and cytosol (Perkins et al., 2015). PrxIV is the only subtype to be identified in the endoplasmic reticulum, which is vital for its antioxidant role governing protein processing protection (Rhee et al., 2012; Yamada and Guo, 2018). The only 1-Cys structural subtype is PrxVI, and this enzyme resides in the cytosolic and lysosomal compartments in cells (Fisher, 2018).

Because of the highly conserved antioxidant function in reducing peroxide agents, the extensive bodily coverage of Prxs allows them to play a significant role in obesity-induced oxidative stress and complications. In mice with PrxV knockout on high-fat diet, progression to obesity and enhanced adipogenesis was far more likely than mice without the gene knockout. Mice with PrxV treatment saw decreased levels of PPARγ and C/EBPα, two markers that indicate an adipogenic state (Kim et al., 2018; Hammarstedt et al., 2018). Another study induced hepatic steatosis in a HepG2 cell line with treatment of FFA to simulate an obese state *in vitro*. Inducing PrxV overexpression ameliorated hepatic steatosis progression by inhibiting lipogenesis associated protein, SREBP-1 and RAS, and FFA-induced mitochondrial ROS generation (Kim et al., 2020). A follow-up study in mice revealed that the mechanism of PrxV in achieving this role appears to be through phosphorylation of AMPK pathway (Kim et al., 2020). Two separate studies have also implicated PrxIV and PrxVI in having a protective role against steatosis by targeting mitochondrial ROS generation (Yamada and Guo, 2018; Lee et al., 2019).

Another study found that PrxIII was expressed at high levels in 3T3-L1 mature adipocytes (Huh et al., 2012). Subsequently, PrxIII knockout (KO) in mice produced a substantial increase in adipocyte hypertrophy and fat mass compared to wild-type mice. The PrxIII KO mice were observed to have increased levels of mitochondrial ROS generation, adipogenic markers, aP2, CCAAT/enhancer binding proteins (C/EBPα), and peroxisomal proliferator activated receptor γ (PPARγ), and protein carbonylation. The study also demonstrated decreased levels of adiponectin, alteration of mitochondrial biogenesis, and other antioxidant enzymes in mitochondria. The PrxIII KO mice were identified to have impaired glucose metabolism and insulin resistance (Huh et al., 2012). Although the mechanism to how PrxIII regulates adipocyte function has yet to be established, this study provides ample data to strongly suggest therapeutic implications for PrxIII in obesity.

Similarly, recent study also identified PrxVI as a vital component leading to an early stage of T2DM with diabetic dyslipidemia in PrxVI KO mice (Pacifici et al., 2014). In the KO mice, elevated levels of triglycerides and VLDLs were identified; a hallmark indicator of adipocyte dysregulation in obesity (Makhoul et al., 2011). Similarly, the PrxVI KO mice exhibited increased levels of pro-inflammatory cytokines, TNFα, IL-1β, IL-10, and IL-6, from adipose tissue. Altogether, this study exemplifies PrxVI in protecting against progression to T2DM with diabetic dyslipidemia (Pacifici et al., 2014). PrxIV is another subtype implicated in its protective role against diabetes due to stimulation of enhanced insulin secretion (Mehmeti et al., 2014). Overall, the mechanisms responsible for mediating the antioxidant actions of Prxs against obesity are somewhat undiscovered or undefined due to the relative recency of many studies involving Prx. However, there is growing evidence linking some Prx subtypes as key protective mechanisms against obesity-induced inflammation and comorbidities.

### N-Acetylcysteine (NAC)

N-Acetylcysteine (NAC) is an antioxidant that serves to reduce ROS through both direct and indirect effects (Mokhtari et al., 2017). The direct effects revolve around its’ ability to react with hydroxyls, nitrogen dioxide and carbonate (Samuni et al., 2013). The indirect effects arise from NAC being derived from a conditionally essential amino acid, L-cysteine and subsequently provides the cysteine for the formation of glutathione, a well-established potent physiological antioxidant (Kerksick and Willoughby, 2005). However, the oral bioavailability of glutathione is controversial (Allen and Bradley, 2011; Richie et al., 2015). Therefore, NAC is marketed as an over-the-counter oral antioxidant supplement while being utilized in many treatment protocols and clinical studies with relatively low adverse effects (Schmitt et al., 2015).

The role of NAC in obesity has yielded promising results across countless studies. Given that obesity is characterized by elevated levels of oxidative stress and inflammation has made NAC a profound target for research to minimize the progression of obesity and associated co-morbidities (Manna and Jain, 2015; McMurray et al., 2016). Recent *in vitro* studies have been able to demonstrate a beneficial role in attenuating biomarkers associated with adipocyte differentiation pathways such as PPARγ and C/EBPβ in 3T3-L1 preadipocyte cells (Calzadilla et al., 2011). The reduction in these biomarkers was shown to be equal to glutathione level elevation through NAC supplementation, reducing ROS, that upregulate the activity of PPARγ and C/EBPβ (Lee et al., 2009; Calzadilla et al., 2011; Pratt et al., 2019). This allows for speculation that supplementation of NAC during the progression of obesity could reduce adipogenesis through elevation of glutathione level content. Furthermore, *in vivo* studies revealed that supplementation of NAC in murine models that were fed a high fat diet to induce obesity, reduced triglyceride and cholesterol content within the liver by reducing PPARγ levels amongst other genes involved in hepatic storage and metabolism of lipids (Ma et al., 2016). Studies have given promising evidence that NAC may normalize levels of hepatic malondialdehyde and superoxide dismutase (SOD) providing further insight that there could be a role in preventing obesity associated co-morbidities of the liver including NASH (Thong-Ngam et al., 2007; Korou et al., 2014).

A major factor revolving around obesity is the oxidative stress associated inflammation present across various metabolic tissues that has shown to be linked to insulin resistance, atherosclerosis, and ischemic strokes (Manna and Jain, 2015; Assari and Bazargan, 2019). The plausible efficacy of NAC in counteracting obesity associated inflammation and apoptosis that contributes to progression of metabolic disorders is attributed to NAC’s ability to reduce inflammatory cytokines and apoptotic factors while increasing antioxidant components (Samuni et al., 2013; de Andrade et al., 2015). These anti-apoptotic effects are elicited by the increased levels of SOD, catalase, glutathione peroxidase and activity of peroxynitrate which serve to reduce ROS (Zaragoza et al., 2000; de Andrade et al., 2015). *In vitro* studies uncovered further contributions to the reduction of inflammation and apoptosis by NAC reducing levels of vascular cell adhesion molecule 1 (VCAM-1), TNF-α, IL-6, IL-8, Ikβ kinase and subsequent activation of NF-κB, which have roles in multiple inflammatory cascades (Samuni et al., 2013; de Andrade et al., 2015; Sun et al., 2019). The capability of NAC to reduce inflammatory pathways and ROS generation may allow for improvement in insulin sensitivity in obese individuals that begin to progress to the development of T2DM (Fulghesu et al., 2002; Lasram et al., 2015; Shen et al., 2018). This was evidenced by recent studies that revealed treatment with NAC improved plasma insulin levels, increased insulin sensitivity across multiple tissues and increased motor activity in murine models of obesity (Lasram et al., 2015; Shen et al., 2018). The increase in motor activity could be attributed to NAC’s ability to improve insulin sensitivity in skeletal muscle tissues as well as possibly preventing the decline of skeletal muscle sodium potassium adenosine triphosphatase (Na^+^/K^+^ ATPase) activity allowing for slowed muscle fatigue (McKenna et al., 2006). Both of these contributions could indicate a theoretical route of research to determine if any correlation exists between NAC and increased total daily energy expenditure in obese individuals which may be combined with proper diet, decreasing the total degree of inflammation by reducing adiposity and fat mass. Furthermore, NAC was shown to improve insulin sensitivity and glucose utilization in hyperglycemia induced mice *via* high sucrose diet as well as in human volunteers during a hyperglycemic clamp (Ammon et al., 1992; Diniz et al., 2006). These studies reinforce the plausible link between NAC and improving insulin sensitivity to glucose which may provide insight into improvement of glucose utilization within obese individuals that have progressed to developing T2DM (Straub et al., 2019).

The current cumulative line of evidence strongly suggests that NAC may have a role in being utilized as a cardioprotective agent by reducing hyperglycemia induced oxidative damage to cardiac myocytes and cardiac remodeling (Chen et al., 1985; Dludla et al., 2017). This reduction in oxidative stress attenuates a progressive loss in cardiac efficiency and cardiac fibrosis that would have progressed to more severe clinical outcomes prior to intervention (Seddon et al., 2007; Talasaz et al., 2013). Data from multiple studies indicated that NAC elevated levels of SOD and glutathione in cardiac myocytes, while reducing levels of TGF-β, lipid hydroperoxides and various biomarkers for oxidative stress (Haleagrahara et al., 2011; Talasaz et al., 2013; Dludla et al., 2017). Obesity and T2DM both exhibit a characteristic feature of hypercoagulable states through chronic inflammation associated with obesity and dysregulation of platelet aggregation (Anderson and Weitz, 2010; Blokhin and Lentz, 2013; Widjaja et al., 2019). Studies have uncovered that NAC may have a potential role in reducing the likelihood of thrombus generation through elevation of platelet glutathione levels and nitric oxide (Girouard et al., 2003; Martinez de Lizarrondo et al., 2017; Craver et al., 2020). The elevation in platelet glutathione ameliorated platelet ROS preventing oxidative constituents to induce coagulation cascades (Wang et al., 2016). It was also indicated that NAC may exert antagonistic effects toward Von Willebrand factor, a necessary component of coagulation cascades, reducing the risk of thrombosis and stroke (Wang et al., 2016; Martinez de Lizarrondo et al., 2017). Through elevating levels of nitric oxide, NAC may have a possible role in regulation of high blood pressure in obese individuals *via* nitric oxide mediated vasodilation (Zicha et al., 2006; Gibson et al., 2011). NAC’s cardioprotective abilities also correlate to its ability to reduce the expression of malic enzyme, fatty acid synthase and other enzymes involved in biosynthesis of triglyceride and cholesterol biosynthesis pathways (Lin and Yin, 2008; Korou et al., 2010; Korou et al., 2014; Ma et al., 2016). This cardioprotective ability may indicate that NAC may play a role in prevention of atherosclerosis.

### Vitamin E

Vitamin E (VitE) primarily describes a group of eight compounds known as tocopherols and tocotrienols where each group consists of α, β, γ, and δ tocopherol/tocotrienol compounds (Colombo and vitamin, 2010; Rizvi et al., 2014). Each of the eight compounds, has shown to elicit some degree of antioxidant properties. However, of all the compounds the constituent α-tocopherol (αT) possesses the highest physiological concentrations (Baxter et al., 2012). It has been shown extensively that αT acts as an antioxidant through its chromanol ring, allowing it to scavenge and remove ROS preventing oxidative damage (Ahsan et al., 2015). Through their lipophilic characteristics, αT serves to protect polyunsaturated fatty acids present in membrane phospholipids and lipoproteins to stabilize cellular membranes (Kuznetsov et al., 1993; Wallert et al., 2014; Galmes et al., 2018). Many studies have suggested a positive correlation between αT and attenuating inflammation, clinical disorders and promoting immune system functionality (Galmes et al., 2018; Lewis et al., 2019). This research along with VitE being widely available, obtained solely from the diet particularly from vegetable oils, has peaked interest into incorporating αT supplementation into many treatment protocols of various clinical disorders including metabolic syndrome where individuals are shown to have lower levels of αT (Montonen et al., 2004; Mehmetoglu et al., 2011). This may indicate that the development and progression of obesity related metabolic syndrome could in part be attributed to reduced levels of αT (Lira et al., 2011).

Recent *in vivo* studies in models of obesity and human studies have indicated that αT supplementation was capable of attenuating inflammatory processes by reducing the expression of IL-6, TNF-α, malondialdehyde and C reactive protein while increasing antioxidant constituents (Patrick and Uzick, 2001; Wong et al., 2017). Furthermore, αT may also act to inhibit pathways that are activated by ROS such as p38 MAPK (Schett et al., 2008; Thalhamer et al., 2008; Wang et al., 2011; Wong et al., 2017). These pathways tend to be elevated in obese individuals, various inflammatory pathologies and are diminished with αT supplementation (Wong et al., 2017; Donohoe et al., 2020). Administration of αT in obese individual induces differentiation of macrophages toward an M2 phenotype that exhibits anti-inflammatory effects (Alcala et al., 2015; Ray et al., 2016). Thus, αT may have a plausible role in reducing inflammation mediated by ROS-induced oxidative damage and exert effects on immune system functionality (Lee and Han, 2018).

The ability of αT to reduce total cholesterol and triglycerides has yielded promising results in metabolic syndrome patients and in prevention of obesity associated atherosclerosis (Upston et al., 2003; Wong et al., 2017). These beneficial effects elicited by αT are mediated through the reduction of low density lipoprotein (LDL), high density lipoprotein (HDL) and total cholesterol levels (Upston et al., 2003; Kirmizis and Chatzidimitriou, 2009; Wong et al., 2017). In murine models of diet induced obesity, αT diminished the degree of hepatic steatosis and circulating triglycerides indicating a plausible role in the prevention of atherosclerosis and NASH, associated with obesity (Kawanaka et al., 2013; Alcala et al., 2015). Cumulative evidence across diabetic individuals, and various animal models indicate that αT is able to reduce lipid peroxidation within cellular membranes and reduce overall lipid content in circulation (Kuznetsov et al., 1993; Wiseman et al., 1995; Eder et al., 2004). This same reduction of lipids can also be seen within adipocytes as αT may serve a role in shifting the metabolic profile of obese individuals toward normalcy. These deviations within adipocytes in obese individuals compared to non-obese individuals includes an elevation of matrix metalloproteinase activity and collagen deposition (Sun et al., 2013; Lauhio et al., 2016). The accumulation of collagen prevents adipocyte expansion and may contribute to the phenotype and inflammation seen in obesity (Unal et al., 2010; Jaoude and Koh, 2016; Lin et al., 2016; Wong et al., 2017). However, supplementation with αT promoted adipocyte differentiation and expansion indicating that αT may play a role in downregulating pathways associated with collagen accumulation and abnormal adipocyte growth to attenuate the pathogenic obesity phenotype (Kim et al., 2007).

Cumulative studies have indicated that αT supplementation reduces biomarkers of lipid peroxidation in T2DM patients and improve insulin sensitivity (Wu et al., 2007; Thalhamer et al., 2008; Murer et al., 2014; Niki, 2015). The elevation in insulin sensitivity may be attributed to the anti-inflammatory action of αT in visceral adipose tissue, by reducing the level of p38 phosphorylation, which is associated with many inflammatory responses and insulin resistance (Wu et al., 2007; Wong et al., 2017). Through scavenging of ROS, αT may serve to ameliorate the impact that persistent hyperglycemic conditions have on promoting ROS production and inflammatory cascade activation (Alcala et al., 2015; Wong et al., 2017). Thus, there may be a role linking αT’s effects on inflammatory cascades that may lead to the attenuation of the diabetic phenotype (Lee et al., 1999).

The actions of αT have shown to attenuate production of ROS and activity of protein kinase C (PKC) pathways associated with cellular cascades that promote proliferation and differentiation (Martin-Nizard et al., 1998). These cascades have shown to be affected by αT through increasing the expression of biological compounds that inhibit PKC resulting in the downregulation of a multitude of cascades (Engin, 2009). Among these cascades, αT exhibits an inhibitory effect on the proliferation of vascular smooth muscle cells that may contribute to CVD diseases such as atherosclerosis (Nakashima et al., 1998), indicating that αT may have a plausible role in cardio protection (Engin, 2009; Rizvi et al., 2014). Evidence has highlighted that αT serves a role in cardioprotective mechanisms through prevention of retinal vascular disease in hyperglycemic states associated with T2DM patients by inhibition of these PKC mediated pathways (Engin, 2009). αT may also serve to attenuate platelet aggregation, stimulate prostacyclin release, and elevate nitric oxide synthase (Rizvi et al., 2014). Moreover, αT reduced the systolic blood pressure through increasing nitric oxide synthase activity in murine models of hypertension (Tran and Chan, 1990; Rizvi et al., 2014). Thus, the elevation of prostacyclin and nitric oxide synthase uncovers a plausible cardioprotective mechanism against hypertension as both are potent vasodilators (Moncada and Vane, 1979; Ahmad et al., 2018). Evidence accumulated across *in vitro* studies suggest that αT may lower the levels of TNF-α induced stimulation of intracellular cell adhesion molecule (ICAM-1) and VCAM-1 (Kirmizis and Chatzidimitriou, 2009; Cook-Mills et al., 2013; Cook-Mills, 2013; Rizvi et al., 2014). This downregulation may indicate diminished platelet aggregation and plausible reduction in the risk for atherosclerosis and thrombosis (Nakashima et al., 1998; Rizvi et al., 2014).

### Heme Oxygenase-1

Heme oxygenase-1 (HO-1) has been established as a stress-inducible enzyme that possesses cytoprotective, anti-inflammatory and antioxidant properties (Nath, 2006; Araujo et al., 2012). Other functions include its role in the rate limiting step for heme degradation as it exerts an indirect antioxidant effect in this manner through the degradation products (Nath, 2006). Heme is a potent pro-oxidant that serves to increase production of ROS seen in many pathological states (Wu et al., 2019). HO-1 degrades heme through a series of catabolic steps that yield products including biliverdin, carbon monoxide (CO) and free iron (Gozzelino et al., 2010). These effects have sparked interest into multitudes of studies for incorporation into therapeutic regimens for a plausible impediment of disease progression in various inflammatory diseases, CVD and metabolic dysregulation associated with obesity (Durante, 2003; Pae and Chung, 2009; Bereczki et al., 2018).

The importance of functioning HO-1 has been shown by *in vivo* HMOX1 gene KO murine models (Poss and Tonegawa, 1997; Chan et al., 2011; Ayer et al., 2016). Studies have shown that induced deficiency of HO-1 results in chronic inflammatory states with elevated levels of pro-inflammatory cytokines that are similar to the levels in various inflammatory diseases (Poss and Tonegawa, 1997; Chan et al., 2011; Lenoir et al., 2017). The anti-inflammatory effects elicited by HO-1 under normal conditions can be attributed to its ability to degrade free heme. In excess, free heme has the capability to contribute to the pathology of various inflammatory diseases through destabilizing cellular membranes, damaging organelles and DNA (Kumar and Bandyopadhyay, 2005; Sparkenbaugh et al., 2015). Biliverdin, CO and free iron produced from degradation of heme have been shown to elicit anti-inflammatory actions through a variety of different cascades (Peterson et al., 2020). CO is able to reduce pro-inflammatory cytokines attributing to the toxicity of free heme including TNF-α, IL-1β and IL-2 through its actions on MAPK and NF-kβ pathways (Kruger et al., 2006; Wang et al., 2009; Burgess et al., 2010; Paine et al., 2010; Chan et al., 2011). This reduction in IL-2 may contribute to most of the anti-proliferative actions associated with CO signaling (Pae et al., 2004; Pae and Chung, 2009). Biliverdin may also regulate its anti-inflammatory actions through multiple mechanisms that involve the inhibition of NF-kβ and enhancing production of anti-inflammatory constituents (Pae and Chung, 2009). The free iron that is released from heme degradation possess inflammatory and cytotoxic characteristics (Dutra and Bozza, 2014). However, to compensate for the elevation in free iron from heme degradation, HO-1 upregulates ferritin synthesis that is capable of storing free iron preventing possible initiation of otherwise harmful inflammatory cascades (Balla et al., 1992; Pae and Chung, 2009). Through the suppression of pro-inflammatory mediators and antioxidant actions, HO-1 and the products of heme degradation are able to reduce inflammation and possibly diminish the phenotypic expression associated with obesity.

Through the reduction in proinflammatory mediators directly and indirectly, HO-1 may contribute to improving insulin sensitivity in diabetics, reducing adipogenesis and the probability of CVD development. Recent studies indicate that the reduced adipogenesis and adipocyte dysfunction may be attributed to the regulation of HO-1 mediated Wnt/β-catenin pathways that act to reduce expression of PPARγ and C/EBPα (Siersbaek et al., 2010; Cao et al., 2012; Khitan et al., 2014). Biliverdin, produced *via* HO-1 degradation of heme, may contribute to normalizing adipocyte function by exerting regulatory effects on PKC pathways (Wegiel and Otterbein, 2012; Khitan et al., 2014). By limiting adipogenesis, adipocyte proliferation and differentiation, HO-1 is able to reduce the inflammatory response within adipocytes promoting a more stable microenvironment (Cao et al., 2012; Wagner et al., 2017). This is due to smaller adipocytes being more responsive to insulin and release higher concentrations of adiponectin compared to larger adipocytes (Peterson et al., 2019). The improvement of insulin action can be attributed to the effects of HO-1 attenuating the expression of insulin resistance mediators including TNF-α, IL-1β and increasing the levels of adiponectin (Kim et al., 2008; Burgess et al., 2010; Cao et al., 2012). Adiponectin is a hormone that is released from adipocytes, that possesses anti-inflammatory characteristics and confers insulin sensitivity (Li et al., 2008). The actions of adiponectin resemble those of HO-1, that decreases the expression of TNF-α, IL-1β, NF-kβ signaling as well as other inflammatory mediators to subvert this pathological phenotype (Kim et al., 2008; Burgess et al., 2010; Aprahamian and Sam, 2011). Furthermore, adiponectin and HO-1 both serve to act on inhibiting AMPK pathway, which is profound in obesity associated inflammation (Hosick and Stec, 2012; Sodhi et al., 2015; Smith et al., 2016; Achari and Jain, 2017). By inducing adiponectin upregulation, HO-1 is able to work in concert with adiponectin involving AMPK cascades to improve insulin sensitivity, attenuate adipogenesis and progression of NASH (Burgess et al., 2010; Hwang et al., 2013; Sodhi et al., 2015; Smith et al., 2016; Peterson et al., 2019).

Further elucidating the importance of HO-1 in CVD associated with obesity is the therapeutic use of statins that have been shown to promote the induction of HO-1 to prevent atherosclerosis (Heeba et al., 2009). The ability of HO-1 to reduce ROS and pro-oxidant constituents diminishes the risk for oxidation of LDL providing cardioprotective mechanisms against generation of atherosclerotic plaques (Cheng et al., 2009). These plaques elicit higher levels of HO-1 induction that can likely be attributed to physiological response to prevent plaque progression, thrombus generation and activation of inflammatory cascades in vascular endothelium (Cheng et al., 2009; Durante, 2011). Moreover, HO-1 may attenuate vascular inflammation by reducing VCAM levels, apoptotic factors, release of inflammatory mediators from mast cells and upregulating anti-apoptotic factors (Takamiya et al., 2002; Kruger et al., 2006; Chan et al., 2011). The cardioprotective mechanisms provided through induction of HO-1 may be attributed to not only the direct actions of HO-1 but also the indirect actions mediated through heme degradation products CO and biliverdin (Chan et al., 2011; Hosick and Stec, 2012; Ayer et al., 2016). CO may activate soluble guanylyl cyclase cascades leading to vascular relaxation and diminishing platelet aggregation to prevent subsequent thrombus formation (Furchgott and Jothianandan, 1991; Cheng et al., 2009; Hosick and Stec, 2012; Ayer et al., 2016). The actions of CO and biliverdin also seem to mediate pathways acting on vascular smooth muscle cell proliferation in response to injury associated from the pathological microenvironment produced from obesity mediated inflammatory responses (Ollinger et al., 2005; Beck et al., 2010). Through the actions of CO and biliverdin, HO-1 inhibitory effect may attenuate the development and progression of multiple vascular diseases associated with metabolic syndrome and obesity (Frismantiene et al., 2018). The plausible contribution of HO-1 in prevention of CVD, NASH, insulin resistance, inflammation, apoptosis, oxidation and adipogenesis provide insight into the importance of utilizing HO-1 and associated pathways in therapeutic regimens to reduce the morbidity and mortality associated with obesity phenotypes.

### Polyphenols

Polyphenols are a type of antioxidants that are commonly found in diets such as cranberries, red wine, green teas, and many others (Fraga et al., 2019). Although the direct mechanism of action of polyphenols is not well understood, many studies have shown that polyphenols attenuates the obesity phenotype and related comorbidities. Polyphenols has been shown to affect the gut microbiota and stimulate signaling pathways that promote fatty acid β-oxidation, mobilization of adipose tissue through lipolysis, lowering of body weight and fat mass by increasing fat use and energy expenditure through thermogenesis induction, adipose apoptosis, satiety, and an increase in basal metabolic rate (Castro-Barquero et al., 2018). Polyphenol’s inhibitory actions include the downregulation of adipose differentiation and adipogenesis, buildup of triglyceride, and chronic obesity-related inflammation.

Cumulative lines of evidence have demonstrated the beneficial role of polyphenol, found in cranberry extract, to improve diet induced obese phenotype in murine model. The study comparing a diet with high fat/high sucrose and high fat/high sucrose with cranberry extract revealed that polyphenols lowers total weight gain, visceral adipose tissue weight, hepatic triglyceride accumulation, and plasma cholesterol levels (Anhe et al., 2015). Also, mice given cranberry extract had similar fasting glycaemia but with lower fasting insulinaemia, which implies that polyphenols accentuate insulin sensitivity (Anhe et al., 2015). In addition to cranberry extract, the polyphenols present in pomegranate extracts have also been reported to prevent and treat obesity. Previous studies have reported that the pomegranate extract could decrease the level of lipids in the blood with significant anti-inflammatory activities. Recent *in vitro* study has also demonstrated that the polyphenols contained in pomegranate extracts in combination with probiotics induces synergistic effects that substantially reduces the triglyceride levels and intracellular lipid increase (Sorrenti et al., 2019). The study showed that *in vitro* treatment with pomegranate extract and probiotics improved the expression of fatty acid synthase, adiponectin, adipogenic markers, PPARγ and SREBP, as well as inflammatory cytokine, IL-6.

Parallel to the availability of cranberry polyphenols, resveratrol is found in red wine and it undergoes rapid metabolism in the small intestine and by the gut microbiota, leading to low plasma bioavailability and complex outcomes (Galiniak et al., 2019). Resveratrol has been shown to increase endurance during exercise by expanding air capacity and oxygen consumption and also increase insulin sensitivity of visceral white adipose tissue in mice (Springer and Moco, 2019). Consumption of resveratrol alters the composition of gut microbiota by increasing symbiotic bacteria like *Bacteroidetes* and decreasing opportunistic pathogens like *Escherichia coli*. The favorable stimulation of *Bacteroidetes* by resveratrol decreases the levels of trimethylamine-*N*-oxide (TMAO), which is associated with chronic diseases like obesity (Schugar et al., 2017). A fecal transplant of resveratrol-fed mice to obese mice improved their glucose homeostasis. Another study demonstrated that resveratrol’s protein interaction stimulates mitochondrial biogenesis and increases the use of lipids while decreasing glycolysis in the muscle and liver by the deacetylation of PGC1α, leading to an increase in energy expenditure (Lagouge et al., 2006). Concomitantly with protein interactions, resveratrol increases lipolysis and decreases adipogenesis by the inhibition of PPARγ and SIRT1 (Houtkooper et al., 2012). Resveratrol’s function as an antioxidant in redox cycling leads to the activation of Nuclear factor-erythroid 2-related factor-2 (Nrf2), increase in SOD, and reduction in BMI, blood pressure and body weight (Springer and Moco, 2019).

Recent advances have suggested the beneficial role of curcumin, a polyphenol antioxidant, in ameliorating obesity phenotype. In vitro evidence suggests the ability of curcumin in the browning of white adipocytes by increasing the protein levels of hormone-sensitive lipase and p-acyl-CoA carboxylase, augmenting lipolysis (Lone et al., 2016). Curcumin has been largely implicated in attenuating insulin resistance, hyperglycemia, hyperlipidemia and comorbidities associated with obesity (Aggarwal, 2010). The cumulative line of evidence suggest that curcumin can modulate various targets involved in obesity and metabolic diseases including, suppression of NF-κB and its regulation inflammatory cytokines (Singh and Aggarwal, 1995), IKK (Aggarwal et al., 2006), JNK (Wang et al., 2009). Studies in murine models also demonstrated the effectiveness of dietary curcumin in lowering triglycerides, cholesterol and phospholipid levels (Rao et al., 1970; Babu and Srinivasan, 1997). Apart from that, bergamot has also been shown to illicit antioxidant properties and ameliorate obesity and associated comorbidities. Bergamot is rich with flavonoids and phenolic compounds that have been shown to improve dyslipidemia and systemic inflammation in patients with metabolic syndrome (Musolino et al., 2019). The mechanistic action of bergamot is mediated by its compounds, bruteridin and melitidin, which bind the catalytic site of HMG-CoA reductase causing inhibition of cholesterol synthesis by replacing its endogenous substrate HMG-CoA (Leopoldini et al., 2010). The antioxidant property of bergamot also stimulates the growth of beneficial gut microbiota. Studies have provided preclinical proof of concept that induction of bergamot may improve the phenotypical and morphological features of NASH along with reduction in adipose tissue inflammation and inflammatory cytokines, IL-6 and TNFα, hypoadiponectinemia, insulin resistance and dyslipidemia (Musolino et al., 2020).

Epigallocatechin-3-gallate (EGCG) found in green tea is the most dominantly studied catechin. Although EGCG is known for its anti-cancer effects, studies have demonstrated a significant role in ameliorating diabetes. In the context of T2DM, EGCG promotes glucose homeostasis and inhibits lipogenesis and gluconeogenesis in the liver in murine models (Li et al., 2018). EGCG further attenuates the diabetic phenotype by improving the wound healing process through lowering of macrophage accumulation and inflammation (Zhang et al., 2018). When treated with EGCG, insulin resistance along with adipose differentiation is also lowered (Khan and Mukhtar, 2018). In the context of lipid metabolism, EGCG stimulates β-oxidation and lipolysis and downregulates lipogenic enzymes and lipid emulsification (Huang et al., 2014).

### Carotenoids

Carotenoids are 40-carbon molecules found in red, yellow, and orange fruits and vegetables and are subdivided into carotenes and xanthophylls (Langi et al., 2018). Carotenes differ in structure from xanthophylls by the absence of oxygen groups, while xanthophylls may have multiple oxygen groups and are more soluble in water (Moran et al., 2018). The complexity of their actions on lipid membranes is due to its unique action of each carotenoid to various compositions of membranes (Johnson et al., 2018). In the small intestine, carotenoids are emulsified into micelles and their absorptions are facilitated by cell surface proteins (Bohn et al., 2017). After absorption, carotenoids are packaged into chylomicrons for transport to the liver and then released into blood. Carotenes are usually packaged into LDL, while xanthophylls are typically packaged into HDL (Wang et al., 2007). Both types of carotenoids are known for their functions in vision, but growing evidence suggests their anti-obesity activities (Coronel et al., 2019).

Studies have demonstrated the roles β-carotene play in adipose differentiation as a vitamin A precursor. β-carotene is cleaved by β-carotene oxygenase 1 (BCO1) into retinal and then rapidly metabolized to transcriptionally active retinoic acid, which is a type of vitamin A (von Lintig, 2012). In cell culture models, retinoic acid inhibits the expression of adipogenic transcription factors like PPARγ, leading to a decrease in adipogenesis (Schwarz et al., 1997). A rat study demonstrated that vitamin A reduce obesity, but only when supplemented to rats with mature adipocytes. When vitamin A was administrated in newborns, adipocyte proliferation was promoted, while supplementation to mature adipocyte led to loss of weight (Coronel et al., 2019). Relatedly, a human study with 29,000 participants revealed that elevated serum β-carotene levels are associated with lower cardiovascular and heart disease (Huang et al., 2018). Other human studies have demonstrated that higher β-carotene is associated with lower incidence of metabolic syndrome (Beydoun et al., 2019) and lower body weight (Berry et al., 2012) (PMID: 22396202). An analogous study showed that higher serum levels of β-carotene are related to lower BMI and subcutaneous adipose tissue in children (Canas et al., 2017).

Similar to carotenes, xanthophylls like fucoxanthin found in seaweed elicit physiological effects against the obesity phenotype and related diseases. Fucoxanthin is processed into fucoxanthinol in the gastrointestinal tract and then amarouciaxanthin A in the liver (Asai et al., 2004). The proportion of the metabolites of fucoxanthin differ in that more fucoxanthiol is found in the visceral organs like the lungs, heart, and kidneys, while amarouciaxanthin A is more preferentially found in adipose white tissue (Hashimoto et al., 2009). *In vivo* study with fucoxanthin and its metabolite’s actions against the characteristics associated with obesity, demonstrated reduction of serum and hepatic levels of triglycerides and inflammatory cytokines like PGE_2_, nitric oxide, IL-1, and TNF-α (Sakai et al., 2009; Hu et al., 2012). The study also demonstrated an increase in HDL levels, while LDL receptor levels decreased (Beppu et al., 2012). Furthermore, the administration of fucoxanthin led to decrease in both mRNA expression of fatty acid synthase and blood leptin levels (Beppu et al., 2013). Further studies have shown that fucoxanthin upregulates uncoupling protein-1 (UCP-1) in white adipose tissue and β3-adrenergic receptor (Adrb3), leading to increased thermogenesis, fatty acid oxidation, energy expenditure, and weight loss (Maeda et al., 2005). Fucoxanthinol and amarouciaxanthin accentuates these anti-obesity actions of fucoxanthin in later stages of adipocyte differentiation by suppressing PPARγ expression and glucose uptake (Kang et al., 2011).

### Superoxide Dismutase

Various forms of mammalian SOD are characterized by where the enzyme is found and what metal it is catalyzed by. SOD1 and SOD3 are associated with copper and zinc, while SOD2 requires manganese (Zelko et al., 2002; Fukai and Ushio-Fukai, 2011). Despite the differing compartmentalization of each forms of SOD in the mitochondria, cytoplasm and extracellular matrix, they all share the function of neutralizing ROS (Miao and St Clair, 2009). Since all SODs are endogenous enzymes, SOD murine trials are conducted through mimetics like 4-Hydroxy-2,2,6,6-tetramethylpiperidine-N-oxyl (tempol), nanoformulated SOD (NanoSOD), and hydrodynamic injections of SOD3 plasmids. Although SODs are known for their essential role in handling oxidative stress, recent studies suggest a crucial role in ameliorating the obesity phenotype.

Mimetics of SOD have been shown to attenuate the obesity phenotype. Recent *in vivo* study demonstrated that mice supplemented with tempol in their diet weighed less than control mice (Samuni et al., 2010). In parallel, a recent study demonstrated that *in vivo* treatment of NanoSOD in high fat diet fed mice improved the levels of plasma triglyceride, liver triglyceride, and hepatic lipid accumulation (Perriotte-Olson et al., 2016). The anti-obesity effects of SOD were demonstrated when mice received hydrodynamic injections of SOD3 plasmids (Cui et al., 2014). The mice induced with SOD3 showed improvement in body weight which was similar to mice fed a regular chow, as compared to high fat diet fed mice (Cui et al., 2014). Concomitantly, the administration of SOD3 led to smaller epididymal, inguinal, and perirenal white adipose tissues in comparison to the same diet without SOD3 (Cui et al., 2014). Furthermore, the study showed that mice fed a high fat diet with adenoviral SOD3 vectors had lower liver weight, levels of triglycerides, cholesterol by weight, and quantity of non-esterified fatty acids, compared to mice fed a high fat diet (Cui et al., 2014). The study provided a conclusive evidence that the induction of SOD3 improved glucose tolerance and insulin resistance in high fat diet fed mice (Cui et al., 2014).

Several studies have demonstrated that SOD analogs have anti-obesity effects in the molecular level by influencing metabolic pathways and related enzymes. In a cell culture of adipocytes, tempol lowered the cellular level of PPARγ and PPARα, and this led to the downregulation of fasting-induced adipose factor (FIAF) (Samuni et al., 2010). PPARγ and PPARα positively regulate FIAF in the liver and skeletal muscle during the fasted state and can lead to adipogenic outcomes (Dutton and Trayhurn, 2008). In a similar study, mice administered with NanoSOD had markedly decreased levels of fatty acid metabolic enzyme Carnitine Palmitoyl Transferase and a lipogenic *de novo* enzyme fatty acid synthase (Perriotte-Olson et al., 2016). This study also revealed the downregulation of the ERK1/2 signaling pathway by NanoSOD. The ERK1/2 pathway can lead to obesity-linked inflammation by the activation of NF-κB (Hennig et al., 2006). In conjunction, the levels of CPT1α, CPT1β, PGC1α, PGC1β, and UCP2, which are mRNA genes associated with energy expenditure and lipolysis, were higher when mice were given SOD3 (Cui et al., 2014).

Inflammation by itself or caused by excess macrophage is intricately linked with obesity, and the induction of SODs alleviates these outcomes. Studies have shown that mice fed a high fat diet and administered NanoSOD, showed improvement in obesity-related macrophage accumulation levels and inflammatory markers like TNFα, MCP1, and MMP12 improved (Perriotte-Olson et al., 2016). In a related study, mice that were fed a high fat diet had crown-like signs of macrophage infiltrations, while mice that were given a high fat diet and injected with SOD3 had minimal signs of infiltrate (Cui et al., 2014). Activated *F4/80^+^/CD11c^+^* macrophages form these crown-like structures and leads to the remodeling and expansion of adipose tissue (Sun et al., 2011). The mRNA levels of obesity-related inflammation genes like F4/80, TNFα, CD11c, MCP1 and IL6 were lower in these SOD3-administered mice and hepatic *de novo* lipogenesis genes like SREBP1c, fatty acid synthase and Scd1 were higher in mice that were just given a high fat diet (Cui et al., 2014).

## Conclusion

This review aims to highlight the therapeutic potential of seven antioxidants and their mechanistic actions in ameliorating the obese phenotype and associated comorbidities including cardiovascular diseases, NAFLD/NASH and T2DM. We demonstrate that obesity propagates cellular oxidative stress and the activation of a multitude of inflammatory signaling cascades that exacerbate the pathophysiological condition. Obesity mediated induction of oxidative stress promotes a shift from physiological homeostasis to favor physical profiles associated with metabolic syndrome. This shift leads to perturbations that include alterations within the phenotypic expression of adipocytes, cardiovascular damage, insulin resistance and abnormal accumulation of triglycerides playing key roles in the morbidity and mortality associated with obesity. Such pathological modifications promote disequilibrium within the redox state disturbing the functionality of antioxidant defense mechanisms. These alterations allow for the generation and sustained action of oxidative free radicals leading to cytotoxic, inflammatory, and apoptotic cascades to be continually initiated. The typical non-obese phenotype allows for scavenging of the oxidative radicals reducing the overall potential for damage. However, the loss of this defense contributes to developing the chronic inflammatory state associated with obesity. Therefore, the roles of various antioxidants were addressed for plausible therapeutic applications to improve clinical outcomes.

The contribution of antioxidants was investigated to determine their efficacy in attenuating the dysfunctional phenotype. Given the ease of availability of these antioxidants as dietary constituents and over-the-counter supplements enables individuals to reap the benefits at minimal cost. This offers an optimal potential therapy for the obese population to slow the development of the obese phenotype. Not surprisingly, the expression of these antioxidants seems to be reduced across *in vitro* or *in vivo* models and in obese individuals. This provides speculation that widespread oxidative free radicals may be a major contributor to the progression of the pathophysiology associated with obesity and the potential need for antioxidant supplementation in the obese population.

**Figure 2:**
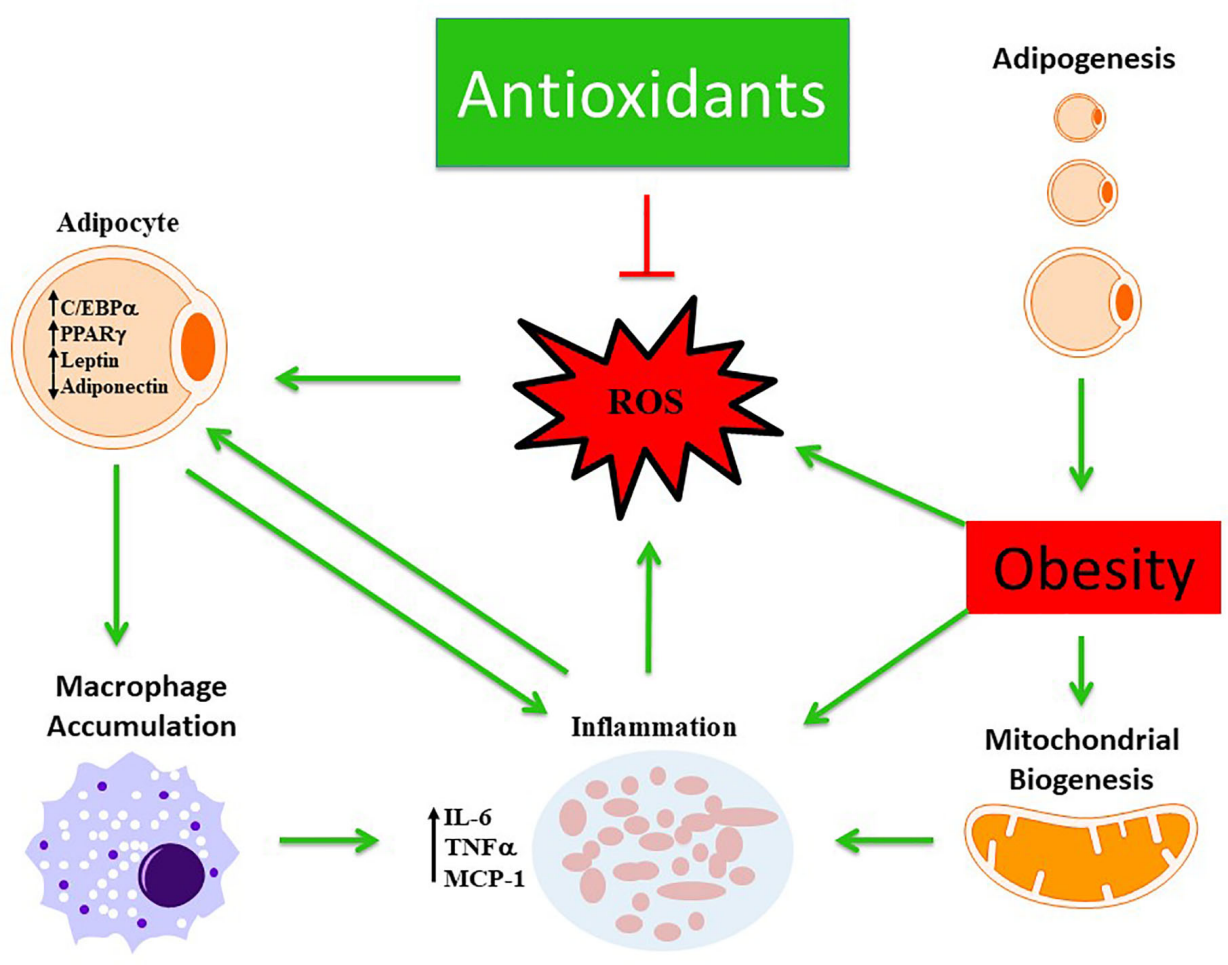
Schematic representation demonstrating the beneficial role of antioxidants in modulating obesity phenotype. Obesity mediates excessive ROS production and inflammation which is further exacerbated by altered mitochondrial biogenesis and activation of macrophages from dysfunctional adipocytes. The antioxidant defense mechanism scavenges ROS and ameliorates obesity phenotype.

The actions of these antioxidants have a hallmark trait of being able to scavenge ROS reducing oxidative stress (**Figure 2**). Each individual antioxidant has its own characteristic mechanism to counteract the pathological environment based on their physical and chemical properties. For instance, the lipophilicity of αT and carotenoids enable them to elicit more beneficial effects within lipid membranes and lipoproteins compared to others. Some of these antioxidants were able to act through indirect and direct mechanisms allowing for the potential to act across various pathways such as HO-1, NAC, various polyphenols and carotenoids. Although there is a long list of endogenous and exogenous antioxidants that have been investigated previously to scavenge free radicals, however, the mechanistic action of each antioxidant is distinct in terms of the pathways they inhibit or stimulate. The specific properties of each antioxidant, listed in this review, provides insight into uncovering a link between which obesity induced metabolic dysfunction they are individually able to respond to optimally, hence these antioxidants were chosen for review. In the case of hypertension, it could be argued that many of the antioxidants may serve a cardioprotective role that is equivalent to each other. However, for hypertension and hyperglycemic conditions one may benefit more from antioxidants that promote insulin sensitivity and lowered blood pressure such as HO-1. Furthermore, many of the antioxidants may serve a role working in concert with one another promoting a synergistic effect across multiple signaling and inflammatory cascades that may attenuate obese phenotypes more-so than one individual antioxidant.

Primarily, the mechanistic actions of each individual antioxidant favored normalization within the inflammatory responses encouraging the progression toward non-obese phenotypes. By acting through inflammatory cascades such as PKC and NF-kβ, these antioxidants serve to reduce the levels of various inflammatory mediators including TNF-α, IL-1 β, VCAM-1, and IL-6 (Carlsen et al., 2009; Kirmizis and Chatzidimitriou, 2009; Burgess et al., 2010; Rizvi et al., 2014; Lee and Han, 2018). Furthermore, antioxidants restore the shift in redox state during altered thermogenesis under diseased metabolic conditions (Wood Dos Santos et al., 2018). The antioxidants, like polyphenols and carotenoids, through induction of thermogenesis, improves the expression of thermogenic markers like UCP1, hence improving the overall mitochondrial function and redox imbalance (Wood Dos Santos et al., 2018). In addition, the activity of proliferative and differentiative cascades within adipocytes were improved leading to ameliorated expressions of PPARγ and C/EBPα (Schwarz et al., 1997; Calzadilla et al., 2011; Cao et al., 2012; Khitan et al., 2014; Springer and Moco, 2019). These effects resulted in a multitude of improvements including enhanced insulin sensitivity, adipocyte functionality, reduced circulating triglycerides, adipogenesis and fat mass (as summarized in **Table 1**). This infers that each antioxidant has the capability to attenuate the development and progression of associated comorbidities through their own mechanisms. Thus, indicating that there may be a strong relationship between antioxidant action and alleviating obesity induced oxidative damage and metabolic dysfunction. The various roles that antioxidants possess may provide a key to unlocking the optimal therapy for the pathophysiology of obesity. More research is required to determine the efficacy of antioxidants working in concert with one another and with current pharmacotherapy in preventing/reverting associated comorbidities of obesity.

**Table 1:** Summary of mechanistic action of antioxidants in ameliorating obesity phenotype and associated comorbidities.

Antioxidant	Source	Pathway/Mechanism	Anti-Obesity Phenotype and Related Comorbidities	References
**Peroxiredoxin**	Endogenous Enzyme	Inhibition of PPARγ and C/EBPα	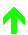 Adiponectin 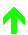 Insulin sensitivity 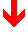 Adipogenesis 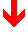 Protein carbonylation	(Huh et al., 2012; Mehmeti et al., 2014; Pacifici et al., 2014; Hammarstedt et al., 2018; Kim et al., 2018; Yamada and Guo, 2018; Lee et al., 2019; Kim et al., 2020)
		Phosphorylation of AMPK	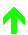 Fatty acid oxidation 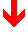 SREBP-1 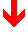 FAS 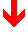 Lipid accumulation	
		Inhibition of IL-6, IL-10, and TNFα	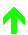 Insulin secretion 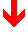 Diabetes 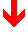 Dyslipidemia	
**N-Acetylcysteine**	Over-the-counter oral supplement	Inhibition of PPARγ and C/EBPβ	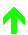 Lipolysis 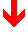 Adipogenesis 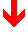 NAFLD/NASH	(Girouard et al., 2003; McKenna et al., 2006; Carlsen et al., 2009; Calzadilla et al., 2011; Haleagrahara et al., 2011; Schmitt et al., 2015; Ma et al., 2016; Pratt et al., 2019; Sun et al., 2019)
		Inhibition of NFkB	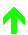 Insulin sensitivity 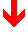 Inflammatory cascades	
		Promotes skeletal muscle Na^+^/K^+^ ATPase activity	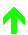 Motor activity	
		Activates cardiac Superoxide dismutase and glutathione	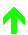 Cardiac efficiency 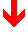 Cardiac fibrosis	
		Upregulates platelet glutathione and Nitric Oxide	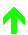 Vasodilation 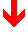 Thrombogenicity	
		Downregulates malic enzyme and fatty acid synthase	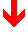 Lipogenesis 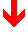 Cholesterologenesis	
		Destabilizes Von Willebrand Factor	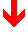 Thrombogenicity	
**Vitamin E**	Vegetable oils, wheatgerm, sunflower, soybean, walnut, over-the-counter oral supplement	Downregulates IL-6, TNF-α, malondialdehyde and c- reactive protein	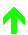 Insulin sensitivity 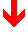 Inflammatory cascades	(Boscoboinik et al., 1991; Cook-Mills, 2013; Alcala et al., 2015; Lin et al., 2016; Wong et al., 2017; Lee and Han, 2018)
		Inhibition of p38 MAPK pathways	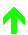 Insulin sensitivity 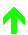 Adipogenesis 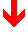 Inflammatory cascades 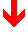 Oxidative cascades 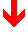 Collagen deposition and fibrosis	
		Upregulates M2 macrophage phenotype	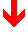 Inflammatory response	
		Inhibition of PKC pathways	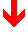 Vascular disease 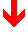 Thrombogenicity 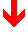 Inflammatory cascades	
		Upregulates nitric oxide synthase and prostacyclins	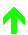 Vasodilation	
		Downregulates LDL, HDL and cholesterologenesis	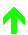 Lipid Mobilization 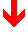 Vascular disease 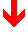 Hepatic steatosis	
		Downregulates ICAM-1 and VCAM-1	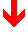 Vascular disease 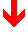 Thrombogenicity	
		Downregulates Matrix Metalloproteinases	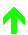 Adipocyte expansion 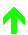 Insulin sensitivity 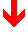 Inflammatory response	
**Heme** **Oxygenase–1**	Endogenous enzyme	Degrades free heme	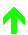 Liberation of CO and Biliverdin 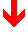 Inflammatory response 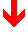 Oxidative cascades	(Burgess et al., 2010; Gozzelino et al., 2010; Chan et al., 2011; Cao et al., 2012; Sodhi et al., 2015; Peterson et al., 2020)
		Inhibits MAPK, NF-kβ, PKC	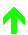 Vasodilation 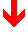 Inflammatory response 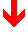 Adipogenesis 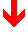 Thrombogenicity	
		Upregulates ferritin synthesis	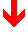 Inflammatory response	
		Inhibition of PPARγ and C/EBPα	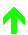 Lipolysis 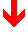 Adipogenesis	
		Downregulates TNF-α, IL-1β, IL-2	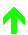 Insulin sensitivity 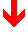 Inflammatory response	
		Upregulates adiponectin	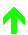 Insulin sensitivity 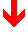 Inflammatory response 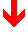 Adipogenesis	
		Reduces oxidized LDL, VCAM-1, mast cell degranulation	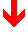 Vascular disease 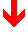 Inflammatory response	
		Upregulates AMPK cascades	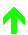 Lipid Mobilization 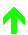 Insulin sensitivity 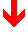 Vascular disease 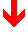 Hepatic steatosis 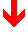 Lipogenesis	
		Activates soluble guanylyl cyclase	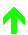 Vasodilation 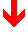 Thrombogenicity	
**Polyphenol**	Cranberry, red wine, green tea, pomegranate extract, curcumin, bergamot	Deacetylation of PGC1α	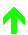 Mitochondrial Biogenesis 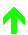 Lipid Mobilization 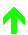 Energy expenditure	(Lagouge et al., 2006; Houtkooper et al., 2012; Fraga et al., 2019; Springer and Moco, 2019)
		Inhibition of PPARγ and SIRT1	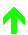 Lipolysis 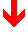 Adipogenesis	
		Activation of Nuclear factor-erythroid 2-related factor-2 (Nrf2)	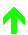 Superoxide dismutase 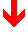 Body Mass Index 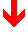 Blood pressure 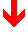 Weight	
**Carotenoids**	Red, yellow, and orange fruits and vegetables	Inhibition of PPARγ	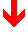 Adipogenesis	(Schwarz et al., 1997; Maeda et al., 2005; Langi et al., 2018)
		Upregulates uncoupling protein-1 (UCP-1) and β3-adrenergic receptor (Adrb3)	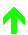 Thermogenesis 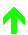 Lipid Mobilization 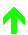 Energy expenditure 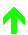 Weight loss	
**Superoxide Dismutase**	Endogenous Enzyme	Inhibition of PPARγ, PPARα, FIAF	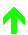 Lipolysis 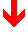 Adipogenesis	(Hennig et al., 2006; Samuni et al., 2010; Perriotte-Olson et al., 2016)
		Decreases levels of Carnitine Palmitoyl Transferase and Fatty Acid Synthase	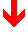 Lipogenesis	
		Inhibition of ERK1/2 and NF-κB	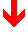 Inflammatory cascades	

This table summarizes the source of each antioxidant along with the mechanism that is modulated with the supplementation of each antioxidant. The mechanistic action of each antioxidant results in amelioration of obesity phenotype and characteristics associated with it. The green arrow (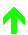) represents upregulation and red arrow (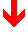) represents downregulation of phenotypical characteristics of obesity in response to antioxidants.

## Author Contributions

Conceptualization: HL and KS. Validation: KS. Writing—original draft preparation: ST, CS, CC, AD, SP, and HC. Writing—review and editing: HL. Supervision: KS and JS. Project administration: KS. Funding: JS. All authors contributed to the article and approved the submitted version.

## Funding

This research was supported by the BrickStreet Foundation and the Huntington Foundation. This research was also supported by National Institute of Health Grant R15 1R15HL150721 (to K.S.).

## Conflict of Interest

The authors declare that the research was conducted in the absence of any commercial or financial relationships that could be construed as a potential conflict of interest.
